# Supplementary material for: Seasonal and social factors associated with spacing in a wild territorial electric fish
Source: PLoS One. 2020 Jun 15;15(6):e0228976. doi: 10.1371/journal.pone.0228976 (PMC7295226; doi:10.1371/journal.pone.0228976)
Supplement: Attachment — Submitted filename: Response to Reviewers_Zubizarreta et al.docx [file pone.0228976.s001.docx]

Revision “***Seasonal and social factors associated with spacing in a wild territorial electric fish***” in accordance with the suggestions that were posed by Reviewer #1.

Below are our answers, point by point to every comment of Reviewer #1, indicating exactly where the changes were inserted (the statements of the Reviewer are presented in black, and our response in red).

Journal Requirements:

When submitting your revision, we need you to address these additional requirements:

1. Please ensure that your manuscript meets PLOS ONE's style requirements, including those for file naming. The PLOS ONE style templates can be found at <http://www.plosone.org/attachments/PLOSOne_formatting_sample_main_body.pdf> and <http://www.plosone.org/attachments/PLOSOne_formatting_sample_title_authors_affiliations.pdf>

Done

2. In your Methods section, please provide additional information regarding the permits you obtained for the work. Please ensure you have included the full name of the authority that approved the field site access and, if no permits were required, a brief statement explaining why.

We added the required information (lines 139-141).

3. To comply with PLOS ONE submissions requirements, please provide methods of sacrifice in the Methods section of your manuscript.

Done, line 187.

4. We note that you have indicated that data from this study are available upon request. PLOS only allows data to be available upon request if there are legal or ethical restrictions on sharing data publicly. For more information on unacceptable data access restrictions, please see <http://journals.plos.org/plosone/s/data-availability#loc-unacceptable-data-access-restrictions>.

In your revised cover letter, please address the following prompts:

a) If there are ethical or legal restrictions on sharing a de-identified data set, please explain them in detail (e.g., data contain potentially sensitive information, data are owned by a third-party organization, etc.) and who has imposed them (e.g., an ethics committee). Please also provide contact information for a data access committee, ethics committee, or other institutional body to which data requests may be sent.

We shared the raw data.

b) If there are no restrictions, please upload the minimal anonymized data set necessary to replicate your study findings as either Supporting Information files or to a stable, public repository and provide us with the relevant URLs, DOIs, or accession numbers. For a list of acceptable repositories, please see <http://journals.plos.org/plosone/s/data-availability#loc-recommended-repositories>.

Done, DOI 10.17605/OSF.IO/65D8A

We will update your Data Availability statement on your behalf to reflect the information you provide.

**Comments to the Author**

1. Is the manuscript technically sound, and do the data support the conclusions?

The manuscript must describe a technically sound piece of scientific research with data that supports the conclusions. Experiments must have been conducted rigorously, with appropriate controls, replication, and sample sizes. The conclusions must be drawn appropriately based on the data presented.

Reviewer #1: Yes

2. Has the statistical analysis been performed appropriately and rigorously?

Reviewer #1: Yes

3. Have the authors made all data underlying the findings in their manuscript fully available?

The [PLOS Data policy](http://www.plosone.org/static/policies.action#sharing) requires authors to make all data underlying the findings described in their manuscript fully available without restriction, with rare exception (please refer to the Data Availability Statement in the manuscript PDF file). The data should be provided as part of the manuscript or its supporting information, or deposited to a public repository. For example, in addition to summary statistics, the data points behind means, medians and variance measures should be available. If there are restrictions on publicly sharing data—e.g. participant privacy or use of data from a third party—those must be specified.

Reviewer #1: Yes

4. Is the manuscript presented in an intelligible fashion and written in standard English?

PLOS ONE does not copyedit accepted manuscripts, so the language in submitted articles must be clear, correct, and unambiguous. Any typographical or grammatical errors should be corrected at revision, so please note any specific errors here.

Reviewer #1: Yes

**5. Review Comments to the Author**

Please use the space provided to explain your answers to the questions above. You may also include additional comments for the author, including concerns about dual publication, research ethics, or publication ethics. (Please upload your review as an attachment if it exceeds 20,000 characters)

Reviewer #1: The authors examine the physio-chemical, hormonal, behavioral and morphological correlates of territory size in a natural population of weakly electric fish. They conclude that territory size varies with oxygen concentration, season, hormonal/gonadal state (in breeding females) and body size (in non-breeding fish). Finally, fish show different affilation seasonally based on sex. This is an impressive data set, one that is rare in teleost fish and unprecedented in electric fish. The data appear carefully collected and statistically analyzed appropriately. The authors test in the field certain relationships that have been thoroughly documented in the laboratory. This paper makes a significant extention of behavioral physiology in the real world of the fish. It will be of great interest to the electric fish community, but also to a broad range of researchers that try to link behavioral physiology with ecology.

Major suggestions:

1. One highlighted conclusion of the paper is that territory size is sexually monomorphic in the non-breeding season, but dimorphic in the breeding season, with females having larger territory sizes than males in the breeding season. The authors speculate that this larger territory size corresponds to a the greater energetic demands of breeding females.

However, this sexual difference only applies when they examine body size-corrected territory size (DNN/body length). Several issues:

a) the authors should be more explicit about why they used body-size corrected territory size for this comparison, but not for other analyses. If it is important to size correct in this comparison, wouldn’t it be important in the general statistical analyses?

Thank you very much for this comment. We first need to clarify that absolute territory size is not sexually dimorphic in any season. As Reviewer #1 points out correctly, the difference between sexes in territory size can only be observed during breeding when we evaluate size-corrected territory size. This being said, to answer why we did not use body-size corrected territory size in all analyses it is important to clarify that this study has been carried out in 3 steps of analysis and that we cannot use all the data we got in all the levels of analyses:

Step 1: We used data obtained in field survey 1 by electrical census (in which we have no morphometric data of the actual fish) to explore how DNNs are affected by ecological parameters (O2 for example, Figure 2) and by EOD rate, which can be remotely assessed (Fig. 2). We cannot use corrected individual size here because we do not have morphometrics of the fish in this step of analysis (Field Survey 1).

Step 2: We used GLMs as an exploratory strategy to evaluate which individual characteristics of fish (morphometrics and/or physiology) impact on DNN in both the non-breeding and breeding seasons. As DNN is the “dependent variable” in these models we therefore cannot use DNN corrected by body size as an “independent variable” (Field Survey 2, Tables 2 and 3).

Step 3. We did a third type of analysis justified by the following arguments: from step 2 we know that while in the non-breeding season body size is the only factor influencing territory size (GLM, Table 3), in the breeding season body size no longer matters. Instead, sexually dimorphic hormone levels are relevant during breeding (GLM, Table 2). It is therefore intriguing that during the breeding season territories are sexually monomorphic in size because they actually depend on traits that are different between females and males. That is why in this third step of analysis, we normalized territory size to body size to see if more subtle differences between sexes were revealed by this approach. We also added the analysis of GSI, which is a traditional proxy of sexual maturity and dimorphism. We revealed a novel dimorphic aspect in spacing during the breeding season (Fig. 3). Females use more space relative to their size, and the relative weight of ovaries (GSI), but not of body weight, strongly correlates to DNN.

Thanks to Reviewer #1 comment, we realized that the 3 steps of analysis associated with Survey 1 and Survey 2 were not explicit in Methods, and thus clarified this in lines 218-132 and 252-264. In addition, we used this same style to present Results (lines 286-287, 320-323, 372-379), Figures and Tables (see figure legends and table captions).

b) It is not clear why they corrected for body size using body length rather than body mass. (They collected both these measurements.) Presumably, energetic demands (e.g. metabolic rate) is a function of body mass, not length.

Thank you very much for this comment that points to an omission we made in our original manuscript which is essential to understand our rationale. As body weight and body length are, as expected, strongly correlated (in this study with R=0.91, now added in lines 230-231), we are confident that this enables us to use any of them as indicators of body size. We chose to use body length because: 1) an operational reason: we had more body length measurements than weight measurements assuring a stronger power to the statistical analyses (now explicit in Methods, line 231); and 2) previous reports in electric fish also preferred to report body length as proxy of body size to show its relevance as a main factor influencing EOD amplitude (Caputi and budelli 2006) and male quality (Gavassa 2012, 2013) (cited in discussion, line 479).

They could even analyze the gonad-free body mass as well as the whole body mass to see if part of the apparent seasonal change in territory size is attributable to the relatively large gonads of females during the breeding season. Or even assess whether DNN correlated to absolute size in female to see if there is evidence that the added mass of a large gonad necessitates a larger territory.

Following Reviewer #1 advice, we added the correlation between DNN and body size without gonads (see Fig. 3D, lines 230-231). Once we showed that DNN does not correlate to body length in the breeding season (Step 2. GLM breeding season, Fig. 3B), we then focused on whether relative gonad weight had any relation to DNN (Step 3, correlations shown in Fig. 3C-D). We agree with Reviewer #1 that it was a good idea to show in different ways if the presence of gonads affects the correlation. We changed Figure 3 to include both the relationships between DNN and GSI, and DNN and body weight without gonads. Indeed, the relative weight of ovaries has a significant correlation (Fig. 3C) whereas body weight without gonads did not (Fig. 3D).

c) In looking at the population means for DNN and body length (Table 1), it looks like the dimorphism in size-corrected DNN arises more from the fact that mean female body size changes less (~32%) seasonally than mean male body size changes (~41%)… rather than sexual differences in the seasonal change in DNN. That is, the seasonal change in this ratio is driven more by the denominator than the numerator. In this regard, the seasonal change may be more about demographic changes that behavioral/physiological changes. This, then, begs the question of why mean body size changes seasonally (see below) and why the change in mean body size differs between sexes

The difference between sexes observed by Reviewer #1 may be apparent but it is not statistically supported. The seasonal variation in body length is significant, but this difference was statistically the same between females and males. The mean body length and standard errors of females are highly overlapped with male values within each season. The seasonal variation in body length is highly significant both, for females and for males, and the % of explained variance is 0.34 for females, and 0.35 for males. Another way to visualize that there are no sexual differences in the seasonal variation in body size is through the comparison of seasonal ∆ (mean body length) between sexes (females: 5.85 ± 1.27, males: 6.56 ± 1.34). This being checked, the point is that males increase size more for a same sized territory (Reviewer #1 phrasing) or that females require an extra territory size than same sized males (our version). Two different ways of phrasing the same thing. We added the hypothesis that demographic changes can also be a way of explaining this seasonal change (Discussion, lines 521-528).

2) Because one of the main conclusions (size corrected sex-differences in territorial behavior) depends on seasonal changes in mean body length, the authors should provide some speculation about why this occurs. What happens to the large fish in the non-breeding season? Do they die? emigrate? Conversely, do the large fish drive out the small fish from the study area during the breeding season?

Thank you for this comment. We added a new paragraph with this hypothesis in the Discussion (Lines 521-528). ‘’The fact that individual body size was larger in the breeding than in the non-breeding season, most likely depends on the physical growth inherent to *G. omarorum* life history. However, we cannot assume that the non-breeding and breeding population remain demographically unchanged. Although this study was not conceived to track population seasonal changes, differences in fish size between seasons may reflect demographic changes. For example, large mature fish may displace smaller adults not only to exploit valuable territories for their own use, but also to access sites where offspring have higher survival as observed, for example, in the cichlid *Lobochilotes labiatus* (88).’’

3) The correlation of territory size with 02 concentration is very interesting. Indeed, it is remarkable that fish can be larger in body size, discharge at high rates, respire at high temperature, defend larger territories and grow their gonads--- all very metabolically expensive -- when many of them exist in waters that are <2mg/L O2. But, again, why was DNN not size-corrected, since O2 requirements depend on body mass? Also, why was O2 concentration not simply incorporated as a covariate in the generalized linear model? It would also be interesting to know if EOD rate or GSI (two metabolically expensive traits) correlated with O2 concentration.

Thank you very much for this interesting comment, which motivated several additions to the manuscript both in new findings (e.g. tests for the suggested relationships) and new points discussed. As explained above, we carried out this study with an approach that includes two surveys and three levels of analyses (response to major comment 1). Further, following Reviewer #1 suggestion, we explored in Step 3 of analysis the linear correlation between O2 content and EDO rate, which showed a positive relationship in the breeding season, but not in the non-breeding season (panel included in Fig. 2); and the linear regression between O2 content and GSI, which showed a positive relationship in breeding females, but not males (panels included in Fig. 3). Moreover, the interpretation of these results was added to the Discussion (lines 456-460 and 513-517).

4) Finally, it would help to state if there were any correlations based on the position of the territory within the field site. For example, were territories on the edge of the mat of plants different in size or O2 concentration? Was one sex more likely to inhabit “edge” territories?

As well as Reviewer #1, we expected to find a relationship between O2 levels, the zone of the mat, and fish body size. We actually performed these comparisons exhaustively and found no significant correlations. This is why we did not include this approach in the manuscript.

Minor comments:

Line 144 & 396: The authors synonomize DNN and territory size, given that DNN is a “proxy” for territory size. Please provide a reference that DNN (a linear measurement) is widely recognized as a reliable predictor of territory area. One could easily imagine that a fish might affiliate closely with another fish (small DNN) but still defend a large territory area.

We included a more detailed justification of using DNN as a proxy for territory size, by adding two references (refs 10 and 44, lines 149-152). We also stated that using DNN as a proxy of territory size is a simplified approach (line 148).

Line 268: I don't understand how the population mean of DNN in the non-breeding season could be 1.4m when the mean for males is 1.2 and for females is 1.13 (Table 1). Also the sample sizes in line 268 are different that those in Table 1. Is this a different data set?

As clarified above (major comment a), we used different datasets and thus the data set for Survey 2 is a fraction of the data set for Survey 1. We hope that with the new structure in the Methods section, these differences are clearer now.

Also, it would be helpful to put the mean size-corrected DNN here, since it would show the size-corrected DNN changes less seasonally than absolute DNN because mean body size increases seasonally.

We think this is a very valuable comment. We added size corrected DNN in Table 1. This addition illustrates the lack of sex differences between corrected DNNs in each season. When we put forth the third level of analysis, in which we look at potential sexual differences from a seasonal standpoint, we reveal an important aspect of sexual dimorphism: breeding females have a relatively larger territory than non-breeding females, whereas males do not show seasonal differences. In addition, this comment helped us realize that relative DNN should be dimensionless. Thus, DNN was expressed in cm and changed throughout.

Line 304: It would be helpful to include the correlational structure in E2, GSI and EOD rate, given that all three correlate significantly with DNN. Do relatively large gonads indeed predict high E2 levels? Is there experimental evidence in Gymnotus that E2 causally reduces EOD rate?

Thank you for this comment that we address in the revised version. However, we first need to clarify that EOD does not significantly correlate with DNN neither in the breeding, nor the non-breeding season. As we state in Discussion, in our study EOD rate is not reliable in its influence upon territory size: it did not appear as a significant determinant in the non-breeding GLM and it only presented a marginal correlation to territory in only one of the significant GLM models that we ran in breeding. So, following Reviewer #1’s suggestion, we added the correlation between GSI and E2 levels (lines 385-386).

Line 319 (Table 1) it might also be useful to include raw values of EOD rate, since this would give the reader a sense of how much less active (in the EOD sense) the fish are in the non-breeding season, and thereby require smaller territory for foraging.

Thanks to this suggestion, raw EOD rate values were added in Figure 2, which clearly shows that breeding EOD rate is way higher than in non-breeding. The observation that fish are electrically less active in winter was also included in lines 328-329 of Results.
