# Supplementary material for: Seasonal and social factors associated with spacing in a wild territorial electric fish
Source: PLoS One. 2020 Jun 15;15(6):e0228976. doi: 10.1371/journal.pone.0228976 (PMC7295226; doi:10.1371/journal.pone.0228976)
Supplement: Attachment — Submitted filename: Response to Reviewers_second revision.docx [file pone.0228976.s002.docx]

Revision “**Seasonal and social factors associated with spacing in a wild territorial electric fish**” in accordance with the suggestions that were posed by Reviewer #2. We also included the remaining editorial suggestions made by Reviewer #1.

Below are our answers, point by point to every comment of Reviewers #2 and #1, indicating exactly where the changes were inserted (the statements of the Reviewer are presented in black, and our response in red).

----------------------------------------------------------------------------------------------------------------------- **Reviewer #2**

This manuscript describes seasonal, water quality, and social effects on territory size in female and male Gymnotus. The data are sound, the statistics are appropriate, and the conclusions are supported by the evidence. I do not have any scientific concerns.

I have made some editorial comments to fix minor problems with the text. There are many “run on” sentences - sentences that would be better divided into 2 or 3 more focussed sentences - that I mostly did not edit. Although those sentences can be parsed, the writing in this manuscript can be improved with some attention to simplifying sentence structure.

We thank Reviewer #2 suggestions, which improved the manuscript.

Abstract

Delete this sentence (it is not particularly informative and not completely correct)

29 The expression of territorial behavior in wild species is especially suited for exploring how animals integrate individual traits with dynamic environmental and social contexts.

Done

Divide this sentence into two separate sentences.

32 This species is a seasonal breeder that displays year-round territorial aggression, in which female and male dyads exhibit indistinguishable non-breeding territorial agonistic behavior and body size is the only significant predictor of contest outcome.

We divided the sentence.

Change “bias” to “biased”

(i) the overall spatial arrangement was sexually bias

Done

Add a comma after ‘females’

females (iii)

Done

Eliminate the phrase

45 “probably to meet sexually dimorphic energetic requirements”

We deleted the phrase

The final sentence of the Abstract is very weak. Either eliminate or replace with a final sentence that communicates the main finding of the work.

46 This study provides evidence of seasonal changes in factors correlated with territory size and contributes to the understanding of the mechanisms underlying behavioral plasticity.

We replaced the final sentence of the Abstract.

Introduction

Change “a hint on” to “insights into”

60 provides a hint on how animals

Done

This statement is not true - body size is not THE universal indicator of strength.

Moreover, body size is the universal indicator of physical strength and thus it strongly impacts contest outcome and territory size. Change to: "Moreover, body size strongly impacts contest outcomes and territory sizes."

We deleted the statement

Change “asymmetry” to “asymmetries”

67 both sexes, asymmetry in fighting abilities

Done

Change “(11); while in” to “(11). In”

70 size (11); while in stripped plateau

Done

Methods

Change

112 “is a weakly electric fish, the only one of several electric fish species in Uruguay that is present in the study area. “ to “is the sole species of weakly electric fish present in the study area.”

Done

Change “carried out” to “performed” in each instance

109 was carried out in the 120 achieved by carrying out an [performing] Done

144 we carried out an 150 “study was carried out during” Done

177 was carried out both, during “was performed during both” Done

152 We carried out the Done

202 analyses were carried out according Done

204 were carried out on samples Done

207 were run to establish [run is also colloquial English] Changed

218	was carried out in 3 Done

222	we carried out a linear Done

256	we carried out linear Done

338	comparisons carried out by t-test. Done

“Carried out” is colloquial English. Its primary meaning, as you know, is to transport an item out of a location.

Eliminate “very carefully”

184 plants was very carefully collected

Done

Change

185 "Netting implies lifting plants as well, thus if" to "Netting disturbs plants: if"

Done

The paragraph starting on line 225 “The second step…” is far too long.

.	A new paragraph should start at “To examine the effect…”

.	A new paragraph should start at “To select the most parsimonious”

.	A new paragraph should start at “The third step” and change “third” to “final”

We divided the paragraph as suggested.

Change “effect” to “effects”

234 the effect of individual

Done

Change “rate” to “rates”

285 EOD rate of fish

Done

I have no idea what is meant by this phrase. Please edit or eliminate.

287 (thus, those individuals were not collected).

We eliminated the sentence

Divide this long paragraph at:

350 For males, we found

Done

Change “search for” to “assess” and “of” to “in” and “territory” to “territorial”

372 To search for sexual differences of territory

Done

Change

375 "a more careful analysis separating males from females" to "an analysis in which males and females were separated"

Done

Discussion

Change

430 “contributes to the understanding of the mechanisms underlying behavioral plasticity by reporting for the first time” to “examined”

Done

Change to “we found that”

435 we confirmed our predictions

Done

Change “concentration” to “concentrations”

451 dissolved O2 concentration and

Done

Change

451 "in places with hypoxia" to “in hypoxic environments”

Done

Change “It” to “Gymnotus”

451 It can breathe

Done

The paragraph that begins on 463 “In teleosts, year round…” is far too long.

Divide at 471 “The generation of the EOD”

Divide at 478 “Electric fish use electric communication”

We divided the paragraph as suggested

Please fix the following run-on sentence. Divide into at least two sentences. This is confusing.

483 Dominance status based on EOD rate may be needed to reinforce submission when a subordinate individual is unable to escape from the dominant as in confined laboratory conditions but not in the wild under the population densities observed in this study.

We divided the sentence and clarified the point.

Divide the long paragraph that starts on 489 “While competition for reproductive” at the sentence “Interestingly, a closer” that occurs on line 496

The paragraph was divided as suggested.

Change “crucial” to “powerful” or “strong” and change “mediators” to “modulators”

508 are crucial mediators

Done

Steroid hormones don’t respond to the social environment - they are molecules. Change

Done.

508 "they respond to the social environment and orchestrate integrated responses " to “Hormones are used to orchestrate organismal-level physiological and behavioral responses to social cues.”

Done

----------------------------------------------------------------------------------------------------------------

**Reviewer #1**

Reviewer #1: The authors have thoroughly addressed all previous comments. They have done an excellent job reanalyzing portions of their data sets, clarifying their analyses, and revising their manuscript in response.

I have only a few remaining editorial suggestions, listed below.

Line 70: “body size”. Done
Line 80 “By contrast”. Done
Line 110, comma before “which”. Done
Line 164, no need to use parentheses here. Done
Line 165, “through two electrodes lowered into the vicinity of the animal and connected to an amplifier…”. The sentence was changed
Line 169, probably no need to define Q10 here. We removed the definition
Line 177, “was carried out during both the breeding and non-breeding season”. Done
Line 185, “Netting requires…”. Done
Line 193 “Dissected gonads were …”. Done
Line 218: Data analysis was carried out in three steps. Done
Line 226: “”… whether individual morphometric and physiological traits correlated with DNN… Done
Line 230: “Because body weight and length were…”. Done
Line 244: “… and considered up to second…”. Done
Line 247 “two”. Done
Line 263 “… its sex confirmed”. Done
Line 320 “The second step of the analysis explored…”. Done
Line 327 “Mean adult body size was significantly larger in the breeding season than non-breeding season.” Done
Line 445: Move citation forward. That is “… to analyzed interspecific interactions (66)” Done
Line 459 “… production (70), which can be….”. Done
Line 496: “… body size influence habitat….” (eliminate ‘on’). Done
